# Interaction and Application of Molds and Yeasts in Chinese Fermented Foods

**DOI:** 10.3389/fmicb.2021.664850

**Published:** 2022-04-08

**Authors:** Qilin Yang, Hongli Yao, Shuangping Liu, Jian Mao

**Affiliations:** ^1^National Engineering Research Center of Cereal Fermentation and Food Biomanufacturing, Jiangnan University, Wuxi, China; ^2^Jiangnan University (Shaoxing) Industrial Technology Research Institute, Shaoxing, China; ^3^National Engineering Research Center of Huangjiu, Zhejiang Guyuelongshan Shaoxing Wine Co., Ltd., Shaoxing, China

**Keywords:** fermented food, molds, yeasts, application, interaction

## Abstract

Fermentation is an ancient food preservation and processing technology with a long history of thousands of years, that is still practiced all over the world. Fermented foods are usually defined as foods or beverages made by controlling the growth of microorganisms and the transformation of raw and auxiliary food components, which provide the human body with many beneficial nutrients or health factors. As fungus widely used in traditional Chinese fermented foods, molds and yeasts play an irreplaceable role in the formation of flavor substances and the production of functional components in fermented foods. The research progress of molds and yeasts in traditional Chinese fermented foods from traditional to modern is reviewed, including the research on the diversity, and population structure of molds and yeasts in fermented foods. The interaction between fermenting mold and yeast and the latest research results and application development prospects of related industries were discussed.

## Introduction

Traditional fermented food has a long history and is widely distributed in the world. Many countries and regions have local characteristics of traditional fermented food which is indispensable delicacies on the table, such as Chinese spirits, soy sauce and fermented bean curd, Japanese natto and sake, Korean pickles, Italian salami sausage, Caucasia kefir milk, Turkish Tarhana, African garri, as well as bread, cheese, and yogurt from many western countries (Table 1). Due to the different geographical environments and life styles, traditional fermented foods from different countries or regions has their own characteristics in production form, flavor and nutritional value (Hesseltine and Wang, 1967).

**TABLE 1:** Main classification of fermented food in the world.

Category	Main fermented food	Main distribution areas	References
Alcoholic beverages	Baijiu, *Huangjiu*, Beer, Wine, Sake,	All over the world	Soni and Dey, 2014; Tamang et al., 2016
Grain	Bread, Pasta, Ogi, Tape, Ketan	All over the world	Campbell-Platt and Geoffrey, 1994; Tamang et al., 2016
Beans	Soy sauce, Natto, Douchi, Sufu	Asia	Nagai et al., 2010
Milk	Yogurt, Cheese, Kefir	Europe, North America, Middle East	Campbell-Platt and Geoffrey, 1994
Meat	Sausage, Ham, Suanyu, Smelly, Mandarin Fish	Europe, North America, China	Holck et al., 2017
Fermented vegetables	Pickles, Suancai, Kimchi	All over the world	Tamang and Kailasapathy, 2010b
Fishes	Fish sauce	East Asia, Southeast Asia, Europe	Liu et al., 2017
Rhizome	Gari, Fufu, Cingwada, Tape, Tapai Ubi	Africa, Southeast Asia	El and Montet, 2014
Others	Vinegar, Pixian, Broad, Bean paste, Black bean, Pidan	Asia	Wang and Fung, 1996; Mo et al., 2008; Wang et al., 2016

**FIGURE 1:**
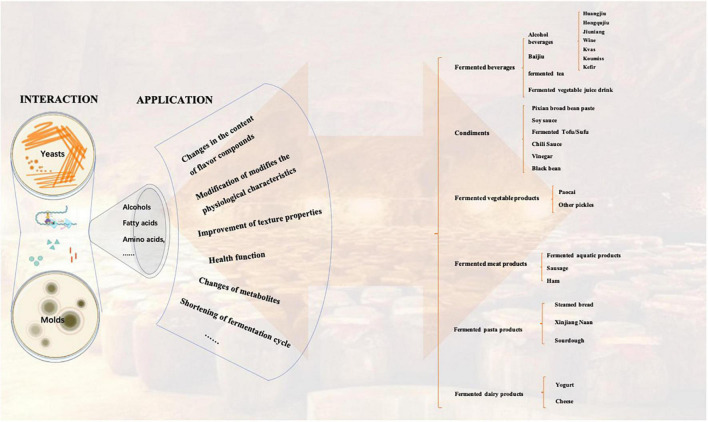
Distribution and classification of traditional fermented foods in China.

As fungi widely used in fermented food, molds, and yeasts play an irreplaceable role in the formation of flavor substances and the production of functional components in fermented food. Traditional fungal fermentation leading to the production of foods and beverages is an ancient bioprocess, but it is still practiced worldwide to date. In Europe, yeast are extensively involved in the brewing of beers and wines. *Penicillium* spp. are used to ripen cheeses and meats. In East Asia, there are a variety of fermented foods produced by molds or yeasts, which have profoundly shaped the eating habits of the locals. *Aspergillus oryzae (Asp*. *oryzae)* is used for brewing soy sauce, miso, sake, ginger, etc. *Monascus* spp. are used to produce red mold rice. Various molds or yeasts play an important role in the production of fermented bean curd, huangjiu, Chinese liquor, tempeh, and so on (Figure 1). *Qu* (Koji) as a fermentation starter is the soul behind these fermented foods, which is composed of cooked grains inoculated with a fermentation culture. The invention of *Qu* embodies the wisdom of the ancient working people without the concept of modern microbiology, which has potentially become an important part of local culture. However, many fermented foods from molds and yeasts have not been widely recognized yet.

Although traditional fungal fermentation has great economic and practical significance in food production, its core problem is that products and fermentative fungi have strong regional characteristics, which also leads to the relative lack of systematic comparison of related research on local characteristics. Therefore, this paper reviews the research history of molds and yeasts from traditional to modern, the diversity, population structure, functional characteristics and the development of analytical technology of molds and yeasts in fermented food, introduces some new research achievements and problems encountered through the role and characteristics of molds and yeasts in all kinds of fermented food, and discusses the fermentation molds and yeasts and related industries. The development prospect of the new technology. It is hoped that this topic can provide a communication platform for the research and application of different types of fermented fungi, and also provide a global perspective for the application of molds and yeasts in fermented food production in China.

## History of Fermented Food Production in China

The wide application of microbial cultures represented by yeast in daily life is one of the characteristics of Chinese culture. In nature, yeasts grow on the surface of fruits (especially berries). These fruits will naturally ferment into wine when they fall to a place that does not leak. Therefore, fruit wine has long existed in nature. Many countries around the world have legends that monkeys love to drink, and there are also historical records of monkeys drinking. To be sure, fruit wines existed long before mankind. Inspired by natural phenomena, mankind has long known the use of fruit to make wine. In addition, in ancient times, humans domesticated a variety of wild animals into domestic animals, and the milk of domestic animals began to be drunk by humans (Tamang and Kailasapathy, 2010a). There are fewer yeasts that ferment lactose in milk than those that ferment glucose in fruits, but there are still enough yeasts to ferment milk into wine. After humans drink livestock milk, they have surplus. The surplus milk is first fermented by lactic acid bacteria into yogurt, and then fermented by yeast into milk wine. Therefore, many nomads will brew dairy wine. It is more complicated to use grain to make wine. The starch in the grain must be hydrolyzed into sugar, and then yeast can ferment the sugar into wine (Farnworth and Edward, 2003). Glucoamylase, which hydrolyzes starch, is rich in grain sprouts. The sprouts are soaked in water, the glucoamylase in the sprouts will hydrolyze the starch into sugar, so the yeasts present on the sprouts will ferment and make it into wine. Under natural conditions, this kind of grain wine will be widely produced everywhere (Steinkraus, 2004). When the Chinese ancestors began agricultural production, the climate was different from now, when the weather was hot and humid, which was suitable for the reproduction of mold. The grains that people store are not only easy to sprout, but also easy to mold. There are many glucoamylases in molds, which can hydrolyze starch into sugar. Over time, humans have begun to purposefully make grains moldy. This moldy grain is *Qu* (for fermented foods, *Qu* is also unique). *Qu* foam can be fermented into wine in water. The ancestors of the Chinese people discovered the mold first, cultivated it and used it first, and then spread it to Asian countries, which occupies an important position in the history of winemaking in the world. Play a great role in promoting social progress (Cheng, 2014).

The famous Japanese microbiologist Kenichiro Sakaguchi believes that China’s creation of *Qu*, the use of mold to make wine, and the promotion of it to East Asia are as important as China’s four major inventions. Why was the use of microorganisms particularly successful in China thousands of years ago? The main reason is that China’s farming era started relatively early, and it is also affected by the monsoon (Monsoon) climate. According to Zhu Kezhen’s research, on the east coast of Eurasia between 20 and 40° north latitude, the summer is controlled by the sinking airflow on the west side of the subtropical high The sinking air absorbs a large amount of water vapor from the warm and wet sea surface, thus bringing abundant precipitation. Formed a humid subtropical climate. Due to the strong contrast between the sea and the land, a unique monsoon climate is formed here. Its notable feature is that it rains in summer and is dry in winter, and the rainfall is concentrated in summer. High temperature and high humidity are naturally suitable for the growth of microorganisms. Therefore, since ancient times, China has referred to the first “C” day after the awn planting as “molding,” and the first day after Xiaoshu as “molding,”0 that is, from June 6–15 to July 8–19. For a period of time, there was a rainy season in eastern China with a long rainy period and concentrated rainfall. During this period, food utensils are prone to mold, so a warning is given in the almanac. At the same time, Zhu Kezhen also conducted an in-depth study of China’s paleo-climate changes and pointed out that in the first 2000 years (from the Yangshao culture in the primitive clan era to the Anyang Yin Ruins in the slave society) in China, the annual average for most of the time was The temperature is about 2°C higher than it is now. The temperature in January was 3∼5°C higher than it is now. Therefore, at that time, the area suitable for the growth of microorganisms would be wider, so most of the “*Qu*” recorded in ancient documents we know today were created in the first 2000 years (Hsieh, 1976).

*Qu* is a fermentation agent containing a large number of living bacteria and enzymes made by cultivating microorganisms with grains or by-products of grain processing. Since the Zhou Dynasty in 841 BC, there have been written records of fermented foods, and most of them used *Qu* as a starter. The earliest form of *Qu* should be germinated and moldy grain seeds (Wang, 2016). After a long period of use and improvement, before the Zhou Dynasty, there was the so-called “*Qu Yi*,” that is, grain *Qu* covered with *Aspergillus* spores, because the strains were relatively high. The simplicity and color are single and bright, indicating that the microorganisms in the *Qu* at this time should be mainly *Asp. oryzae* that produces yellow spores, and of course yeasts are indispensable. Since molds were grown on scattered grains to make *Qu* at that time, they are now called “*San Qu*.” In the Han dynasty, “*Bing Qu*” was popular, because more kinds of microorganisms could multiply inside the “*Bing Qu*,” which made the microbial composition in the *Qu* more abundant. First of all, there are more yeasts. More importantly, *Rhizopus* is easier to grow inside the *Qu* than *Aspergillus*, and bacteria with strong ability to produce lactic acid and decompose proteins are also easier to occupy a place in the *Qu*. Later, wild herbs were added to the *Qu*, which provided the yeast with vitamins and other nutrients, which made it grow more vigorously, so that my country’s unique alcoholic double fermentation process of saccharification and fermentation was formed. Because of the diversity of the microbial flora of *Qu*, not only saccharification and alcohol fermentation, but also the decomposition of protein and fat by other microorganisms, as well as the formation of various biochemical metabolites, make the ingredients in the wine extremely complex and unique (Yang, 2013). The flavor. However, due to the development of *Qu* making technology, especially through the continuous continuation of the “*Mu Qu*” process with fine *Qu*, the utilization effect of *Qu* has been continuously improved. Make *Qu* a means to enrich good bacteria, and make *Qu* a form of long-term preservation of good bacteria. Many excellent pure strains used in modern fermentation industries in China and Japan, many of which are obtained by selecting and breeding after being separated from *Qu* (Zhang, 2001).

In 1929, Wei Yanshou reported a new species of Mucor isolated from fermented bean curd in *Science*. In 1932, Chen Jusheng began to isolate 15 strains of yeast and several species of *Aspergillus* from Nanjing and other places of wine and medicine, and to conduct morphological and physiological studies on them. In the early 1930s, he also contributed to the improvement of my country’s traditional soy sauce brewing process during his stay at the Central Industrial Laboratory. For example, he isolated *Asp. oryzae* with strong protease activity from soy sauce and successfully used it to make pure *Qu* to brew soy sauce, which attracted the attention of domestic brewing circles. In 1937, Jin Peisong observed various *Aspergillus*, *Rhizopus* and yeast isolated from *Qu* from all over China to observe their color under ultraviolet light, which is easy to identify their types. In 1956, Fang Xinfang began to study the classification and important physiological characteristics of Rhizopus isolated from *Xiaoqu*, and determined that *Rhizopus* is the main saccharifying bacteria of *Xiaoqu*. With the development of the fermentation industry, the research on the technology and its core microorganisms is gradually moving toward modernization, mechanization, thoroughbredness and intelligence.

Chinese *Jiu Qu* has a long history, diverse types, unique functions, and each has its own merits (Wan, 2007; Zhu, 2011). The use of *Qu* is a great invention of Chinese ancestors, and it is a precious scientific and cultural heritage of the motherland. “*Shu Jing Shuo Ming*”: “If you make wine, you will only be able to squeeze tillers.” This is the earliest written record of the ancients on the wine making of *Jiu Qu*. The use of *Qu* is the characteristic of Chinese winemaking, and it spreads to Japan, Vietnam and other Asian countries, and it is also the watershed of the world’s eastern and western wine culture. For the core microorganisms in the fermentation process, mold and yeast, and their role in the fermentation process, it is an important subject so far, and there are many mysteries.

## The Discovery of Molds and Yeasts of Traditional Fermented Foods in China

Fermented food contains rich microbial resources because of the open system of the fermentation. After a long period of evolution, a large number of excellent microbial resources have been retained. The study of microorganisms in fermented food is an important way to deepen the understanding of the molecular mechanism of fermentation process. Therefore, in recent years, a great deal of analysis has been performed for the succession and diversity of microorganisms in the fermentation process of traditional fermented food. In fermented food, the application of denaturing gradient gel electrophoresis (DGGE) and high-throughput sequencing (HGS) in traditional pure culture methods has broken the limitation of traditional culture methods, making the microbial community map of fermented food more clear and accurate (Muyzer et al., 1998).

In the past, microorganisms were known mainly through traditional culturable methods, which is to first isolate and purify the microorganisms, and then identify the species of the isolated strains. According to the diversity of yeasts in Italian traditional fermented olives, 117 strains of yeasts were isolated, of which 87 strains belonged to *Saccharomyces cerevisiae* (*S. cerevisiae*), and the rest were *Pichia galeiformis*, *Candida* and so on (Tofalo et al., 2013). However, this method has great limitations, time-consuming and laborious, and can only detect a limited number of microbial species, which cannot accurately determine the microbial composition of the fermentation system. In the past decade, with the wide application of modern molecular microbial ecology technology based on PCR-DGGE technology and high-throughput sequencing technology, people’s understanding of microbial species and microbial succession in the manufacturing process of traditional fermented food has undergone a qualitative leap (Liu et al., 2012).

At present, in the field of traditional fermented food, there are few reports on the integration and analysis of the data generated by different ensemble platforms. With the gradual reduction of sequencing cost and the continuous improvement of sequencing accuracy, the acquisition of abundant accurate data will no longer be the threshold to limit the research. The more important problem that researchers need to solve is how to analyze the vast amount of data deeply and effectively according to biological problems, so as to completely analyze the types and functions of microorganisms in the brewing system. The research progress of mold and yeast composition in microbial community of traditional fermented food is shown in Table 2. The main fungi in traditional fermented food in China are *Rhizopus*, *Mucor, Aspergillus*, *Penicillium*, while *Saccharomyces*, *Candida*, *Coccidioides*, etc. For example, the fermentation bacteria of huangjiu include *Mucor*, *Rhizopus*, *Aspergillus niger* (*Asp. niger*), *Monascus*, *Aspergillus flavus* (*Asp. flavus*), and other molds, as well as yeast such as *Trichosporon* and *Candida*. The dominant fungi (>1% abundance) were *Saccharomyces* and *Aspergillus*, and *Saccharomyces* accounts for 11.34–25.01% of the whole microbiota (Liu et al., 2019).

**TABLE 2:** Research advances in studying microbial community composition and function of traditional fermented foods.

Traditional fermented food		Main fermentation microorganisms	References
Fermented beverages	Shaoxing Huangjiu	*Saccharomyces*; *Aspergillus*, etc.	Bokulich et al., 2016; Bourrie et al., 2016; Zheng and Han, 2016; Cai et al., 2018; Wang et al., 2018; Wei et al., 2018; Gao and Zhang, 2019; Guo et al., 2019; Liu et al., 2019; Tang et al., 2019; Chen et al., 2020; Liu Z. et al., 2020; Nejati et al., 2020
	Wheat Qu	*Rhizopus*, *Rhizomucor*, *Aspergillus*, *Penicillium*, etc.	
	Jiuyao	*Saccharomycopsis*, *Rhizopus*, *Aspergillus*, etc.	
	Hongqujiu	*Wickerhamomyces anomalus*, *S. cerevisiae*, *Monascus* *purpureus*, *Xeromyces bisporus*, etc.	
	Hongqu	*Monascus*, *Rhizopus*, *Asp. niger*, *Asp. flavus*, *S. cerevisiae*, etc.	
	Jiuniang	*R. oryzae*, *R. microsporus*, *Asp. niger*, *Asp. c*and*idus*, *Mucor indicus*, *Mucor circinelloides*, *Saccharomycopsis fibuligera, S. cerevisiae*, *Pichia sp*., *Pichia burtonii*, *Candida glabrata*, *C. metapsilosis*, *C. rugosa*, *C. tropicalis*, *Kodamaea ohmeri*, etc.	
	Wine	*Saccharomyces*, *Kloeckera apiculate, Candida, Metschnikowia, Hanseniaspora, Schizosaccharomyces, Hansenula, Debaryomyces, Zygosaccharomyces* *Hanseniaspora*, *Issatchenkia*, *Rhodotorula*, *Penicillium*, *Cladosporium*, *Botrytis*, *Sporobolomyces*, *Aspergillus*, *Cryptococcus*, *Pichia*, etc.	
	Koumiss	*Kluyveromyces marxianus*, *Kazachstania unispora*, *Dekkera anomala*, *S. cerevisiae*, *Trichosporon asahii*, *Penicillium carneum*, *Pichia membranifaciens*, etc.	
	Kefir	*Kluyveromyces marxianus*, *Dekkera anomalus*. *Kazachstania unispora*, *Kazachstania turicensis*, *Kluyveromyces marxianus, S. cerevisiae*, *Dekkera anomala*, *Asp. Amstelodami*, etc.	
	Baijiu	*Rhizopus, Aspergillus, Mucor, Absidia, Rhizomucor, Penicillium; S. cerevisiae, Pichia, Zygosaccharomyces, Schizosaccharomyces, Saccharomycopsis*, etc.	
Fermented tea	Kombucha; Oolong tea; Pu’er tea; Dark tea (Fu-Brick Tea); Black tea	*Penicillium*, *Aspergillus Saccharomyces, Yarrowia*, etc.	
	Soy sauce	*Aspergillus*, *Zygosaccharomyces rouxii, Candida*, etc.	Marsh et al., 2014; Huang et al., 2017; Li et al., 2018; Rui et al., 2019; Arıkan et al., 2020
Condiments	Vinegar	*Eurotium, Monascus, Asp. Pichia, Saccharomyces*, etc.	Lindner et al., 2008; Nie et al., 2017; Yunping et al., 2018
	Fermented Tofu/Sufu	*Aspergillus*, *Monascus*, etc.	
	Paocai	*Pichia, S. cerevisiae*, etc.	
Fermented vegetable products	Sausage, Panxian Ham, Jinhua Ham, Xuanwei Ham, Suanyu, Smelly mandarin fish, Fermented shrimp paste	*Aspergillus*, *Penicillium* *S. cerevisiae* *Virgibacillus halodenitrificans*, *Asp.s niger*. *Pichia gilliermondii*, etc.	Patra et al., 2016
Fermented meat products	Steamed bread	*S. cerevisiae*, *Cyberlindnera jadini*, *Wickerhamomyces anomalus*, *C*and*ida tropicalisstrain*, *R. oryzae*, *Torulaspora delbruecki*, *C*and*ida humilis*, etc.	Aquilanti et al., 2007; Połka et al., 2015; Duan et al., 2016; Lv et al., 2019; Wang et al., 2019
Fermented pasta products	Sour dough	*S. cerevisiae*, *S. exiguus*, *Candida milleri*, *Pichia norvegensis*, *Hansenul anomala*, *Candida krusei*, etc.	Bai et al., 2010; Ercolini et al., 2013; Zhang et al., 2019
	Yogurt, Cheese	*Aspergillus*, *Galactomyces; Kluyveromyces*, *Torulaspora*, etc.	

In recent years, research on the composition of mold and yeast in the microbial community of traditional fermented foods has developed rapidly. Therefore, the study of its function also lays a foundation for the study of the safety, flavor quality, and nutrition of the corresponding fermented food. The scientific connotation of mold and yeast in traditional fermented food needs to be further analyzed and its application value needs to be further explored.

## Function of Mold and Yeast in Traditional Fermented Food

The fermentation system of traditional fermented food is composed of one or more microorganisms. In the traditional fermentation process, the microorganisms involved in metabolism are uniformly enriched in their respective places of origin to form a complete and complex structural system. As the traditional fermentation process is formed in the natural environment, different regional environment, climate and other factors will make the flavor of fermented food different, and the role of microflora is also different. Fermented food and beverages are organisms produced by the activities of microorganisms, mainly yeasts, molds, and bacteria. Fungi (yeast and mold) play an important role in traditional fermented food (Tamang and Kailasapathy, 2010a).

## Yeast

Yeast plays an important role in the production of many fermented foods. About 21 major genera with several species of functional yeasts have been reported from fermented food and beverages which include *Brettanomyces*, *Candida*, *Cryptococcus*, *Debaryomyces*, *Galactomyces*, *Geotrichum*, *Hansenula*, *Hanseniaspora*, *hyphopichia*, *Kluyveromyces*, *Metschnikowia*, *Pichia*, *Rhodotorula*, *Saccharomyces*, *Saccharomycodes*, *Saccharomycopsis*, *Schizo saccharomyces*, *Torulopsis*, *Trichosporon*, *Yarrowia*, and *Zygosacchartztorius* (Aidoo, 1986; Hesseltine, 1991, 2003; Nout and Aidoo, 2010).

Yeast has a direct or indirect relationship with alcohol fermentation, higher alcohols, esters, organic acids and so on, which has a great impact on the flavor of products. Among them, *S. cerevisiae* is the most widely used in fermented foods such as bread and alcoholic drinks. In the fermentation process, *S. cerevisiae* mainly converts small molecular sugars into ethanol and carbon dioxide, and can also metabolize a small amount of other flavor substances. For example, alcohol fermentation is mainly a process in which yeast converts glucose into alcohol and carbon dioxide. Glucose produces pyruvate through the EMP pathway. Pyruvate dehydrogenase catalyzes pyruvate to acetaldehyde, which is then reduced to ethanol by alcohol dehydrogenase. In the process, other by-products are also produced in addition to alcohol and carbon dioxide, such as glycerol, amyl alcohol, isoamyl alcohol, butanol, isobutanol, and other higher alcohols (collectively referred to as fusel oil) and a variety of esters and so on. Alcohol has a refreshing aroma, higher alcohols have a certain flavor, glycerol has a refreshing sweet taste, these alcohols are also the premise of the formation of esters.

## Molds

There are relatively few molds in fermented food and beverages, including *Actinomycetes*, *Mucor*, *Rhizopus*, *Amylomyces*, *Monascus*, *Neurospora*, *Aspergillus*, and *Penicillium* (Hesseltine, 1991; Hesseltine, 2003; Nout and Aidoo, 2010). The main role of these molds in fermented food is to produce a variety of enzymes (Tamang, 2010). For example, protease (acidic, neutral, alkaline), amylase, glutamidase, pectinase, hemicellulase, and cellulase can use starch, oligosaccharide and monosaccharide as carbon source, and protein, amino acid and urea as nitrogen source. Maltose can effectively induce *Asp. oryzae* to secrete various hydrolytic enzymes, such as *Asp. oryzae* to secrete α-amylase, *Asp. Niger*, and *Asp. nigrum* to produce Glucoamylase and so on. Starchy raw materials are degraded into small molecular sugars such as dextrin, maltose and glucose under the action of amylase and glucoamylase. On the one hand, they promote the growth of bacteria, yeasts and other microorganisms, and further metabolize to produce alcohols, organic acids and other flavor substances. On the other hand, some monosaccharides, oligosaccharides, and polysaccharides that can’t be decomposed increase the nutritional value of the products. Protein raw materials are decomposed into peptides, amino acids and other functional and flavor substances by protease. At the same time, these small molecular substances also contribute to the growth and metabolism of bacteria and yeast (Tamang, 2010). Therefore, deepening the understanding of mold and yeast in fermented food is helpful to promote the progress of fermented food.

## Interaction Between Mold and Yeast in Traditional Fermented Foods

In the production of traditional fermented food, not only the microorganisms are closely related to the environment in the fermentation process, but also the ecological relationship among microorganisms is very complex. The synergism and antagonism among microorganisms have a profound impact on the formation of the final flavor of fermented food and the generation of new substances. With the progress of systems biotechnology, the complex interactions among microorganisms are studied at multi-level by using multi-group technology as an important means. Genomics, transcriptome, proteomics, and metabonomics reveal a series of changes in the life process of microorganisms at the levels of DNA, RNA, protein and metabolites, respectively, which is helpful to a comprehensive and in-depth understanding of the complex interactions among microorganisms. The application of these techniques further reveals the physiological mechanism of interactions among different microbial populations, and provides theoretical guidance for the directional regulation and application of microorganisms. For example, *Aspergillus* was the main strain producing tea fuscin in the early stage, while *Mucor* and *S. cerevisiae* were also involved in the production of tea fuscin in the later stage (Li et al., 2018). *Pseudomonas* and *Aspergillus* are important strains for the production of methoxyphenols (Li et al., 2018). In the production of alcohols, *Aspergillus* was the main production strain in the initial stage, and *Bacillus*, *Cunninghamia lanceolata*, *Lhasa and Listeria monocytogenes* played a role in the later stage. For instance, the succession of *Staphylococci* on cheese skin is mainly driven by biological factors. It can promote the growth of beneficial staphylococci by strengthening specific molds, so as to inhibit the reproduction of potential pathogens in cheese production (Kastman et al., 2016). So the analysis of the interaction of molds and yeasts in traditional fermented food will help to: (1) deeply understand the law of community succession and analyze the fermentation mechanism; (2) find out the source of flavor substances; (3) through microbial interaction, inhibit the growth of harmful microorganisms or the production of harmful metabolites to ensure the safety of traditional fermented food. Deepening the understanding of the interaction between microorganisms in traditional fermented food is helpful to enhance the formation of flavor substances or control the production of bad flavor substances from the source of fermentation.

The fermentation agents currently in use are mainly yeasts and some molds. These microorganisms have evolved over tens of millions of years (Papadimitriou et al., 2015). Through the acquisition and deletion of genes, they can obtain the corresponding niche in the biological environment of fermented food. These characteristic microorganisms in fermented food are separated and used as starters to ensure the stability of the food. However, the microorganisms involved in the natural fermentation process of food are mostly complex and diverse (Smid and Lacroix, 2013). Diverse microorganisms change their own functional characteristics through interactions, thereby changing the species composition and functions of the entire microbial system in the entire fermented food system, which will ultimately affect the quality and safety of fermented food. A detailed understanding of these interactions is a prerequisite for optimizing and controlling the quality of fermented foods. Therefore, clarifying the mechanism of the interaction between microorganisms in fermented foods can help people develop new starters of mixed strains, better regulate the parameters of the fermentation process, and produce stable and excellent fermented foods.

With the analysis of the microbial community structure of a large number of brewed foods, the core microorganisms in the production process of different brewed foods have gradually been identified, and the deep-level microbial interaction has received more and more attention, but there is still a lack of microbial interactions in traditional Chinese brewed foods (Hong et al., 2016). In-depth study of the mechanism of action. Microbial interaction is becoming a new direction for in-depth analysis of the production mechanism of traditional brewed food. At present, researchers are paying more and more attention to the research of co-enzyme mechanism in brewed food, mainly focusing on the traditional fermented food (Nie et al., 2017). Research on interaction mechanism, quality formation, maintenance, and deterioration mechanism under processing conditions (Wolfe and Dutton, 2015). Xu Yan found that the complementary metabolism of yeast and lactic acid bacteria during liquor fermentation is the basis for the formation of sulfur-containing flavor substances (Liu et al., 2017). Liu et al. (2015) found that microbial co-fermentation can significantly increase the concentration of flavor substances in huangjiu. There are a large number of metabolites in huangjiu that can regulate the physiological functions of fungi, β-phenethyl alcohol, tyrosol, chromanol, and farnesol. For example, the fungal metabolite farnesol can inhibit The cell division of *S. cerevisiae* is in the G1 phase and reduces the content of diglycerides, thereby inhibiting the proliferation of *S. cerevisiae*. To perfectly analyze the formation mechanism of the interaction between the two bacteria, it must be from four levels: functional phenotype, metabolic level, transcription level, and gene level In-depth elucidation of the functional complementarity, metabolic interaction and transcriptional interaction between the two bacteria and the identification of key genes (Albuquerque and Casadevall, 2012).

For example, the brewing of huangjiu uses wheat *Qu* and mother of wine as starters to form a double-sided fermentation process at room temperature while saccharification and fermentation, while wine and beer, which are the world’s three major ancient wines, can only be fermented on one side (Chen and Xu, 2013). The core of this process difference is huangjiu (Liu S. et al., 2020). The use of microbial interaction makes the decomposition of raw materials and the formation of flavors proceed simultaneously, realizing the efficient division of labor and coordination of complex community metabolism: (1). The component modules of different sources and functions are functioned by different strains, which facilitates functional partitioning, avoids cross-effects, and does not increase the metabolic burden Completion of complex work at the same time; (2). The degradation of raw materials and the synthesis of flavor are carried out simultaneously, which avoids the inhibition of microbial metabolism due to high sugar content; (3). The degree of fermented alcohol (18–21°) is much higher than that of wine and beer (<14°). The concentration and abundance of substances are significantly improved, and the production efficiency is significantly improved.

Huangjiu brewing is an open and complex fermentation process. The microorganisms involved mainly come from malt, yeast and the production environment (He et al., 2017). The microorganisms include bacteria, molds and yeasts, especially *Aspergillus* and yeast, which participate in the process of huangjiu brewing. The whole process of saccharification and liquefaction, alcohol fermentation and flavor formation play a vital role in the quality of huangjiu. The brewing process of huangjiu is a bilateral fermentation process in which solid and liquid coexist. The decomposition of raw materials and the utilization of substrates are carried out simultaneously. In the whole fermentation process, yeast and *Aspergillus* are the most important microorganisms. The fermentation process involves the growth of microorganisms, the degradation of brewing materials, the consumption of oxygen, the utilization of substrates, and the accumulation of ethanol organic acids. In the process of huangjiu brewing, the main yeast is *S. cerevisiae*, which plays a role in producing alcohol and flavor (Wu et al., 2015). Some studies have found the existence of Pichia in both manual *Qu* and cooked wheat *Qu* using traditional microbial isolation and culture technology. *Saccharomycopsis fibuligera* was found to be an absolute predominance in huangjiu medicine. In the fermentation process, the mold secretes enzymes for saccharification and liquefaction while the yeast is fermented. In brewing, the mold provides abundant enzymes and becomes an essential brewing microorganism. Studies have pointed out that the molds in the huangjiu brewing process are mainly derived from wheat *Qu* microorganisms. Chen Jianyao and others have used traditional microbial separation. Researches have pointed out that *Asp. oryzae* and *S. cerevisiae* coexist in the early stage of white wine fermentation, which is crucial for saccharification and fermentation, ethanol and flavor substances. Importantly, different ratios of *Asp. oryzae* and *S. cerevisiae* have different effects, and multiple strains will affect the diversity of glucoamylase production and thus have a synergistic effect on yeast production of ethanol (Wang et al., 2020).

In general, there are various types of interactions between microorganisms, and the interaction mechanism is complex. The formation of the nutritional and sensory qualities of fermented foods does not only rely on the action of a single microorganism, but also the interaction between different microorganisms and their metabolites, and finally forms a unique fermented food. The study of the interaction between important microorganisms, mold and yeast is of great significance for understanding the fermentation mechanism of traditional fermented foods and the study of filling interactions.

## Application of Microbial Co-Fermentation Regulation in Fermented Food Production

The analysis of microbial community structure of traditional fermented food and the study of microbial interaction laid a good theoretical foundation for understanding its brewing mechanism at the system level, constructing an efficient and controllable mixed strain fermentation system, and realizing efficient and directional production of products. On this basis, the researchers put forward the application of functional microbial co-culture in fermented food production. The advantages of microbial co-culture have been gradually explored and valued by people, especially in recent years, there have been many successful reports on reforming traditional fermentation process, improving product quality and safety, shortening fermentation cycle and so on.

Co-fermentation plays a very positive role in improving the taste and favor of fermented food. For example, different proportions of *Bacillus licheniformis* and *S. cerevisiae* were inoculated into sorghum extract and fermented, and then the volatile components of the product were analyzed. The results showed that co- fermentation had almost no effect on the growth of *S. cerevisiae*, but had a certain inhibitory effect on the growth of *B. licheniformis*. Under the condition of co-fermentation, the amount of ethanol and flavor compounds produced by *S. cerevisiae* increased significantly, including four fatty acids and their corresponding two esters, one terpene, and five aromatic compounds. At the same time, sixteen kinds of flavor compounds in the product were increased by the addition of *B. licheniformis*, which showed that co-fermentation had a positive effect on the flavor of fermented food (Zha et al., 2018).

### Changes in the Content of Flavor Compounds

Compared with single fermentation, the co-fermentation of *S. cerevisiae* Y3401 and *Wickerhamomyces anomalus* Y3604 produced more ethyl acetate and increased the content of other flavor compounds such as β-phenylethanol and phenylethyl acetate (Fan et al., 2019). The co-fermentation of non- *S. cerevisiae* (*Hanseniaspora opuntiae*, *Hanseniaspora uvarum, and Torulaspora delbrueckii*) and *S. cerevisiae* had lower ethanol content and total acidity, higher volatile aroma components, especially higher alcohols and esters, which was an effective way to improve the sensory quality of fruit wine (Hu et al., 2020). Compared with the single fermentation of *S. cerevisiae*, the co-fermentation of *S. cerevisiae* by *Issatchenkia terricola* SLY-4 and *Pichia kudriavzevii* F2-24 had lower volatile acidity and higher content of aroma components, which improved the flavor and quality of wine. At the same time, sequential co-fermentation is more conducive to the improvement of wine flavor and quality than simultaneous co-fermentation, because the content of esters is higher, and the content of C6 compounds, benzene derivatives, higher alcohols, and fatty acids is lower (Shi et al., 2019).

### Modification of Modifies the Physiological Characteristics

The yeast *S. cerevisiae*, which is incapable of synthesizing glucosylceramide (GlcCer), adapted to alkaline and ethanol tolerance conditions after exposure to GlcCer from *Qu* cereal cultured with *Asp. kawachii*, and modifies its flavor profile (Kazutaka et al., 2015). In nitrogen gas-sparging anaerobic culture of *S. sake* Kyokai No. 7, supplementing the basal synthetic medium with phosphatidylcholine enhanced the yeast growth and fermentative activity, whereas adding ergosteryl oleate enhanced alcohol-endurability. Supplementation with both phosphatidylcholine and ergosteryl oleate promoted the yeast growth, fermentative activity and alcohol-endurability of cells (Hayashida and Ohta, 1980). These novel insights demonstrate a new mechanism of cooperation between microbes in food fermentation and a new technical approach for the modification of fermentation.

### Improvement of Texture Properties

Extracellular polysaccharides (EPS) produced by *Lactic acid bacteria* (LAB) and organic acids produced by *Propionic acid bacteria* (PAB) are used to enhance the texture and extend the shelf life of baked products. The extracellular polysaccharide co-cultured with *Weissella confuse* 11GU-1 and *Propionibacterium freudenreichii* JS15 had synergistic effect on wheat dough and bread texture (Tinzl-Malang et al., 2015). The multi starter fermentation system of LAB and yeast was used to obtain new type of acidified goat milk (AGM), which reduced the relative content of free octanoic acid in AGM, promoted the forming of more aroma in AGM, covered up the fishy smell of goat and made AGM have pleasant flavor. It provides a new choice for people who are hypersensitive to milk protein but do not like the goat flavor of goat milk (Huang et al., 2020).

### Health Function

Edible yeast, *Lactobacillus plantarum* (*L. plantarum*) and Mucor were used in traditional fermentation of three food materials with wheat dough, pickled Chinese cabbage and Mao-tofu, respectively. These microorganisms are able to enhance OPPs dissipation in these fermented food materials, and yeast and Mucor are more potent than *L. plantarum* to degrade OPPs (Zhou et al., 2015). Soybean co-fermentation with different microorganisms (*Bifidobacterium*, *B. subtilis*, and *Rhizopus oligosporus*) in a specific order has higher nutritional value than single fermentation (Puri et al., 2015). *S. cerevisiae var. Boulardii* (S.B.) strain has no negative effect on beer aroma, and adding S.B. strain into the mixed starter can improve antioxidant activity and polyphenol content (Angela et al., 2018). When *Seabuckthorn* was co-fermented with *S. cerevisiae* and *Aesculus orientalis*, the content of the ascorbic acid decreased by 14% (Negi and Dey, 2013). The combined action of *Asp. oryzae* MAO103 and *A. oryzae* MAO104 from Meju, which is a traditional fermented soybean starter in Korea, reduced the mutagenic ability of base substitution of aflatoxin and significantly inhibited the production of aflatoxin by *Asp. flavus* (Lee et al., 2016). *L. plantarum* Shanghai brewing 1.08 and *Zygosaccharomyces rouxii* CGMCC 3791 were inoculated into pickled cabbage and radish, and fermented for 8 days. During the fermentation, the content of nitrite in the system decreased continuously, while its content in the natural fermentation group increased at first and then decreased slightly. The results showed that co-fermentation could effectively inhibit the formation of nitrite and reduce its content in sauerkraut (Wu et al., 2013).

### Changes of Metabolites

*S. cerevisiae* SY1 could not produce ethanol in milk fermentation, but the ethanol production was higher when co-cultured with *L. plantarum* ZL1 (Kuda et al., 2016). By using it to hydrolyze the pretreated biomass, engineered *S. cerevisiae* 424A makes to the co-fermentation of xylose and glucose (mainly glucose and xylose) to ethanol, which can reduced the investment cost. Compared with single hydrolysis, co-fermentation (SHCF) can save energy and increase ethanol yield by reducing the inhibition of terminal products (Jin et al., 2012).

### Shortening of Fermentation Cycle

The fermentation cycle of Chinese traditional fermented fish products (CTFPs) co-fermented by *L. plantarum* 120, *S. cerevisiae* 2018 and *Staphylococcus xylosus* 135 was shortened, and the contents of total volatile base nitrogen, trimethylamine, dimethylamine, nitrite, and N-nitrosodimethylamine (NDMA) were significantly lower than those of natural fermentation samples (Liao et al., 2018).

## Conclusion and Perspectives

As a traditional food with a long history, traditional fermented food is widely spread, and has a variety of health functions on human body. The industrialization level of traditional fermented food in all over the world is not high. There is little knowledge and experience in the production of fermented food for reference. At the same time, the product quality is unstable, and there are many potential safety hazards. Mold and yeast, as important microorganisms in fermented food, not only can form complex flora structure in the fermentation process, but also can produce complex and diverse flavor components. Its growth and metabolism process can improve food structure and texture.

Traditional culturable methods and molecular microbial ecology methods are difficult to systematically analyze the structure and function of such complex microbial flora. In the face of such challenges, microbiome technology which is based on microbiology, functional genomics, metabonomics, bioinformatics, and systems biology, has developed rapidly in recent years. It can reveal how the natural inoculation of microbial flora affects the food fermentation process, and also determine the safety, flavor characteristics, the quality and nutritional function of traditional fermented food (WileyBlackwell, 2013).

The study of molds and yeasts in traditional fermented food will be beneficial to analyze their metabolic mechanisms and complex interactions during the fermentation. This review can provide some new opinions for the research of microorganisms in fermented food, and it can also provide some theoretical guidance for the upgrading and transformation of the industrialization of traditional fermented food. Therefore, the application value of complex and diverse molds and yeasts needs to be further explored (Adams and Mitchell, 2002).

## Developing the Resource of Molds and Yeasts

The production technology of traditional fermented food has been inherited for thousands of years, which is also the process of microbial domestication. And the microorganisms in the brewing system may co-evolve with human beings. The physiological and metabolic characteristics of some domesticated microorganisms may be different from those of undomesticated microorganisms in nature (Bing et al., 2014). For example, it was identified for a bifunctional lipase gene of glyceresterase phospholipase with high catalytic activity and substrate applicability in *Rhizopus chinensis* (a common fungus in *Qu*). The application of protein engineering modification greatly improves the thermal stability of lipase, reduces the production cost, and fills the blank of industrial production of lipase (Yu et al., 2009). *ARO8* gene in deficient yeast strains can significantly improve the ability of glucose conversion to β-phenylethanol (Romagnoli et al., 2015). At present, culturable methods are often used to excavate the microbial resources of fermented food. In the future, it is necessary to establish the culturable method of the difficult culture microorganism. Traditional fermented food microbial germplasm resources and gene resources will be deeply explored to provide new microorganisms, enzymes and gene elements for modern biotechnology industry.

## Quality Control and Optimization of Traditional Fermented Food

Using tyrosine as substrate, the *A. oryzae* 3.042 was adaptively evolved, the content of tyrosine in soybean paste fermented by *Asp. oryzae* decreased from 6.49 to 6.14 mg/g (*p* < 0.05). After optimization, its content decreased to 5.67 mg/g (Niu et al., 2018). *Meyerozyma* and *Candida* were added to *Aspergillus* type Douchi so that the content of all amino acids, organic acids and the percentage of unsaturated fatty acids were significantly increased. The results further indicated that the co-fermentation could improve the flavor components of *Douchi* (He et al., 2019). Therefore, the rational use of the interaction between mold and yeast and other microorganisms has important guiding value for the directional and efficient control of microbial flora metabolism, which also has far-reaching significance for the improvement of product quality. In the near future, we believe that the continuous progress and development of mold and yeast in fermented food will help to improve the quality, stability, and safety of fermented food.

## Microbial Interaction Is Becoming a New Direction to Analyze the Mechanism of Traditional Fermented Food Production

Microbial interactions are widespread in nature, especially in fermented foods. The growth of microorganisms also has a certain impact on the formation of flavor substances, such as yeast and mold at the late stage of sausage fermentation, it has been reported that the interaction between yeast and mold, yeast and bacteria has an important influence on the flavor of liquor, and the interaction of these microorganisms endows fermented food with rich taste and mouthfeel. Study of traditional fermented food fermentation of yeast and mold in the process of interaction, cannot only clarify the ebb and flow of yeast and mold in the process of fermentation, also helps to parse and factors which influence the growing phenomenon of interaction mechanism, so as to provide certain guidance for the fermentation process, has important scientific significance and application value.

## Author Contributions

QY carried out the initial literature review and wrote the initial manuscript. JM and SL provided expertise and insight relating to fermented food. HY revised the manuscript and checked it. All authors read and approved the final manuscript.

## Conflict of Interest

SL and JM were employed by the company Zhejiang Guyuelongshan Shaoxing Wine Co., Ltd. The remaining authors declare that the research was conducted in the absence of any commercial or financial relationships that could be construed as a potential conflict of interest.

## Publisher’s Note

All claims expressed in this article are solely those of the authors and do not necessarily represent those of their affiliated organizations, or those of the publisher, the editors and the reviewers. Any product that may be evaluated in this article, or claim that may be made by its manufacturer, is not guaranteed or endorsed by the publisher.
